# Scrolling through adolescence: a systematic review of the impact of TikTok on adolescent mental health

**DOI:** 10.1007/s00787-024-02581-w

**Published:** 2024-10-16

**Authors:** Giulia Conte, Giorgia Di Iorio, Dario Esposito, Sara Romano, Fabiola Panvino, Susanna Maggi, Benedetta Altomonte, Maria Pia Casini, Mauro Ferrara, Arianna Terrinoni

**Affiliations:** Department of Human Neuroscience, Unit of Child and Adolescent Neuropsychiatry, Sapienza University of Rome, Via dei Sabelli 108, 00185 Rome, Italy

**Keywords:** Social media, TikTok, Adolescents, Mental health, Body image, Problematic social media use

## Abstract

Amidst rising concerns about mental health in adolescents, the role of social media (SM), particularly highly visual platforms such as TikTok, has become a growing focus of investigation. In the extant literature, conclusive evidence is limited by the aggregate analysis of very heterogeneous SM platforms. This systematic scoping review examines the relationship between TikTok and mental health in adolescents. Additionally, it aims to highlight potential interactions between typical developmental processes and the unique aspects of TikTok, exploring their relevance to mental health within this demographic. Following PRISMA guidelines, a systematic search across PubMed, Scopus, Web of Knowledge, and PsychINFO yielded 20 studies that met the inclusion criteria, encompassing a total of 17,336 subjects from 10 countries. The retrieved studies analyzed four main topics related to TikTok use, i.e., the overall impact on adolescents’ mental health, risk of problematic use and behavioral addiction, consequences on body image and self-esteem, and possible spreading of mental illness behaviors. While TikTok offers creative opportunities for self-expression and peer connection, the literature raises concerns about its potential negative effects on teens, such as lower life satisfaction, increased risk of “contagion” of certain psychiatric symptoms, and problematic usage patterns. Significant variations in study methodologies and outcome measures were observed. In the current era of evolving SM landscapes, a comprehensive approach informed by developmentally grounded research is critically needed to further understand the interplay between SM and adolescent mental health, providing insights for health authorities and policymakers.

**Supplementary Information:**

The online version contains supplementary material available at 10.1007/s00787-024-02581-w.

## Highlights

TikTok has become a prominent platform for teens to discuss and display mental health issues.

Reviewed articles indicate an overall negative impact of TikTok on youth mental health, although robust evidence is lacking.

Research mainly focused on the risk of addictive use, spreading of mental illness behavior, and decreased self-esteem.

Type of TikTok use and psychological characteristics of users may influence positive or negative mental health outcomes.

**Supplementary Information:**

The online version contains supplementary material available at 10.1007/s00787-024-02581-w.

## Introduction

Adolescence is marked by profound changes, both physical and psychological. While deep brain rearrangements [1] alongside pubertal development occur, adolescents face the difficult challenge of shaping their personality and building their future in an age filled with environmental and social stressors. As such, this developmental phase represents a critical time frame for mental health [2–6], as highlighted by the onset of over half of mental health problems during adolescence and the increased likelihood of symptom persistence into adulthood [7].

In recent times, adolescents have been facing unprecedented circumstances that may have introduced additional mental health challenges beyond those commonly associated to their age, including the COVID-19 pandemic and its consequences on daily life and social interactions [8–13]. However, few years before the pandemic outbreak, rising concerns about adolescents’ mental well-being had been already outlined, with reports indicating a decline between years 2012 and 2016, accompanied by decreases in self-esteem and happiness [14, 15]. Importantly, depression diagnoses among youth in the US surged from 8.7% in 2005 to 11.3% in 2014 [16], while deaths due to suicide for people aged 15–19 showed a staggering 47.5% increase since the year 2000 [17]. Similarly, while youth emergency department visits for non-fatal self-harm were relatively stable in the US before 2008, rates significantly increased thereafter - particularly among females aged 10 to 14 years [18]. Thus, since the early 2010s, accumulating data from different countries has pointed to a worsening in teen mental health, often described as a “mental illness epidemic,” which began several years prior to the COVID-19 pandemic [19–21]. This trend has prompted researchers to delve into the underlying reasons. The burgeoning popularity of smartphones and social media (SM) over the past fifteen years has garnered growing attention in connection with the notable increase in mental health issues among youth observed globally [14, 22–25]. The advent of the SM era has dramatically changed the way many individuals daily interact and create narratives of themselves. Children and adolescents, particularly, represent the most active SM “consumers”, being continuously connected whenever, wherever, and with whomever they want. This unprecedented situation has prompted professionals, policymakers, and stakeholders to carefully examine and monitor the possible impact of SM use on the mental health and well-being of children and adolescents. Confirming the growing scrutiny on the topic, in May 2023, the U.S. Surgeon General issued an official statement, highlighting the potential risks posed by SM to adolescent mental health as an urgent public health issue. The statement included digital education recommendations based on the existing evidence, albeit limited, on the positive and negative impacts of SM on children and adolescents [26].

Undoubtedly, numerous factors are playing a role in the observed population-level trends of declining mental health in teens. Nevertheless, in the research community, the debate concerning the contribution of SM on this issue is igniting. The ubiquity of SM platforms, with estimates suggesting that up to 97% of adolescents aged 13–17 years utilize at least one platform [27], has intensified the discussion. Across different SM, TikTok particularly gained worldwide popularity becoming the second most used platform in this age range (63% of respondents), with 17% using the platform “almost constantly” [27]. Preliminary evidence suggests that the unique features of the different SM platforms, such as TikTok, Whatsapp, Snapchat, and Facebook, are relevant to the possible risks posed to the mental health of their users [28]. TikTok stands out for some distinctive features that may specifically have significant implications for adolescent users [29, 30]. Compared to other platforms, TikTok has an interface that facilitates instantaneous content consumption and creation, fostering a sense of spontaneity and creativity. Moreover, TikTok’s original design, centered around lighthearted and entertaining content, aligns with the preferences of the younger demographic for easily digestible and engaging material. Over time, TikTok has evolved into a unique space where the “Gen Z” (born 1995–2012) feel free to express themselves authentically, without the fear of judgment from older generations, emerging as a digital safe place for uninhibited creativity and the unfiltered articulation of individuality. Further, TikTok was among the first SM to create by itself a customized feed sending audiovisual contents tailored for users, based on their preferences, interests, and current state of mind [31], through the app’s recommendation algorithm [32]. Also, interactions with other users on TikTok mostly occur through user-generated video content being, again, highly personalized [33]. New videos are constantly presented by default on the user’s ‘For you’ page and often experienced as an endless [32], hard-to-anticipate [34] flow of auto-looped videos to swipe through [35], prompting high levels of engagement and commitment, a phenomenon termed as the “flow experience” [36, 37].

The nature of TikTok’s contents has also been pinpointed as a matter of interest in terms of consequences for the mental health of its users. Over recent years, TikTok has exhibited a surge in contents regarding mental illness, body positivity, neurodiversity, and gender identity [38, 39]. Notably, many accounts describing the journeys of users with conditions such as eating disorders (ED), Tourette’s Syndrome, Dissociative Identity Disorder, Attention Deficit-Hyperactivity Disorder (ADHD), and borderline personality disorder received millions of views [40–43]. A large portion of users are exposed to videos related to mental health [44] and such content has the potential spark off social contagion of symptoms of mental distress or even self-diagnosed mental disorders among individuals who are particularly vulnerable to psychopathology [31]. This issue is increasingly observed in youth seeking psychiatric care [29, 45, 46].

Many previous studies have focused on the general impact of SM on adolescents’ mental health but relatively few specifically analyzed the effect of TikTok in this regard. While some of them found detrimental effects in terms of anxiety [47], depression [48], psychological distress [49], disordered eating [50], sleep [51], and wellbeing [52], others suggested no such connection [53, 54] or offered mixed results [55]. Coherently, in a previous umbrella review, Valkenburg et al. [56] concluded that the associations between SM use and mental health are ‘weak’ or ‘inconsistent’ for most of the available evidence. Such contradictory results may derive from the lack of consistency across investigated outcomes [57, 58] or study groups [59]. Moreover, despite the abundance of studies, few have specifically analyzed how the distinctive features of the various SM platforms are possibly related to mental health outcomes, by focusing on multiple platforms simultaneously without considering differences in the approach to content presentation and social interactions offered by each.

Given its extreme popularity among adolescents and the types of contents shared or accessed by teen users, the present systematic review aims to analyze how TikTok use may be connected to mental health outcomes in this age group and deepen our understanding on the relationship between SM and well-being, exploring both positive and negative influences on adolescents’ daily life.

## Materials and methods

**Fig. 1:**
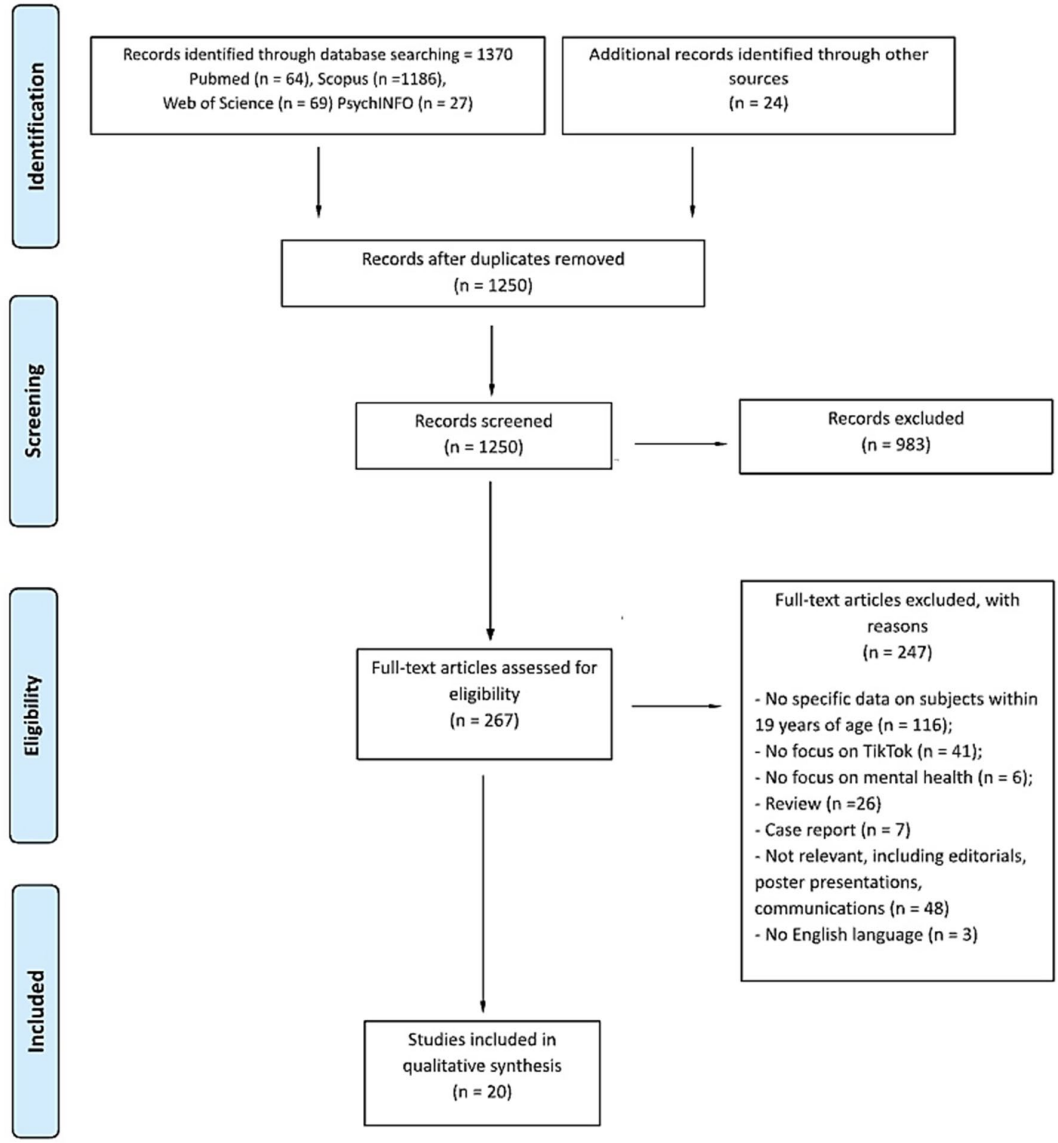
Preferred Reporting Items for Systematic reviews and Meta-Analyses (PRISMA) flow diagram

The present systematic review was conducted in accordance with the PRISMA guidelines for systematic reviews and meta-analyses [60] (See Fig 1).

Studies retrieval and selection process of eligible articles to be included in the current work followed three steps. Firstly, a predefined algorithm was established and used to search for suitable publications in scientific databases of interest. Subsequently, duplicates were removed, and titles and abstracts of the retrieved article were first screened: papers that passed title and abstract screening were further analyzed and screened according to predefined inclusion and exclusion criteria. Finally, data of interest were extracted from the remaining articles.

### Database search strategy

Database search was conducted on the major scientific electronic databases in the field of health, psychology and biomedical science: Pubmed, Scopus, APA PsychINFO, and Web of Science. All studies published up to April 30th, 2024, from the inception of the databases, were scrutinized, without applying any publication year filter. Database search was conducted according to the following search algorithm: (“adolescen*” OR “child*” OR “pediatric” OR “paediatric”) AND (“tik tok” OR “tik-tok” OR “tiktok”) AND (“mental health” OR “mental illness*” OR “psychopatholog*” OR “psychiatr*” OR “conversion” OR “tic disorder” OR “Tourette” OR “dissociative identity” OR “neurodevelopmental disorder” OR “ADHD” OR “attention deficit hyperactivity disorder” OR “suicide” OR “suicide attempt*” OR “self-injury” OR “self-harm” OR “self harm” OR “non suicidal self-injury” OR “body image” OR “eating disorder”).

### Literature search strategy and study eligibility

After article retrieval through the search algorithm, all duplicates were removed, and all articles were considered only once. The remaining publications underwent title and abstract screening to exclude articles that were not relevant to the topic. Subsequently, the selected articles were checked according to predefined inclusion and exclusion criteria.

Inclusion criteria were: (a) studies focusing on the SM TikTok (excluding its equivalent regional names such as Douyin, due to documented differences in their algorithms and characteristics) [61, 62] (b) studies including subjects within 19 years of age (which is the age limit for adolescence, according to WHO) [63] and presenting data specifically on them; (c) studies focusing on aspects related to mental health (including specific neurodevelopmental and psychopathological conditions).

Exclusion criteria were: (a) studies written in languages other than English; (b) case reports, reviews, letters to the editor, commentaries, meeting abstracts, book chapters, dissertations, study protocols, and seminars; (c) animal models; (d) studies that did not include any subject but focused only on content analysis on the SM. Specifically, since our study is centered on the mental health impact at the individual level, it was crucial to focus on research that examined the psychological and behavioral outcomes related to individual characteristics; (e) studies that combined multiple social media (SM) platforms in their analysis, making it impossible to isolate and specifically assess the impact of TikTok.

Besides these exclusion criteria, any other study design was considered, in order to include the highest number of articles focusing specifically on adolescents, TikTok, and mental health. No article was excluded due to unavailability of the full text. References section of articles that survived the application of inclusion and exclusion criteria were checked to search for additional relevant literature: whenever relevant citations were found publications underwent the study eligibility process.

The selection process was carried out by two independent reviewers: in case of disagreement, reviewers discussed their views until a consensus was reached. When necessary, consensus was pursued involving a third reviewer.

### Variables of interest and data extraction

Articles that survived the selection process were then analyzed and information about the following variables of interest was extracted, according to the PICOS (Participants, Interventions, Comparisons, Outcomes, and Study design) system [64]: (a) authors and publication year, (b) type of study, (c) sample demographic characteristics, (d) controls characteristics (when available), (e) data about the SM Use, (f) assessment procedures, (g) evaluated outcomes (wellbeing and mental health indicators), (h) significant findings, (i) quality assessment. Quality assessment of the retrieved publications was performed employing a quality index derived and adapted from the Newcastle-Ottawa Scale (NOS) on a 9-star model [65] (online resources). For further quality evaluation details, see Supplementary Table 1 S.

## Results

From the initial database search, a total of 1370 potentially eligible articles were retrieved; subsequently, duplicates were removed, and a total of 1250 manuscripts were then screened by reading title and abstract. 267 papers survived the first step; inclusion and exclusion criteria were then applied for further screening of the articles: of these, 20 fulfilled all the predefined criteria and were included in the present systematic review. Table 1 shows an overview of the general characteristics of the included manuscripts. Table 1S and 2 S (online resources) display further information about the quality assessment process.

**Table 1:** Synthesis of included studies

References	Design	Subjects		Country	Assessment		Main Findings	Limitation	QA
		*N* (%F)	Age range		Tools	Outcome			
Bucknell Bossexn et al., [74]	Cx	159 (72%)	11–16	Denmark	Self-developed questionnaire.	Gratification motivations.	Passive consumptive behaviors were the most prominent forms of behavior. Gratification of entertainment/affect was the key driver behind passive consumption, participatory and contributory behaviors. Pre-adolescent girls were the heaviest users of TikTok. Individuals who engaged at the contributory level tended to be heavy users.	1) Small sample size 2) Selection bias	4/9
Burke et al., [75]	N	7 (71%)	2–10	Canada	Day-in-the-life methodology and narrative inquiry.	Creativity, Innovation, and Resilience.	The conversion of everyday social skill building, such as children’s continuous engagement with each other via TikTok, into digital play when in-person activity is restricted shows children’s creativity and capacity to adapt in the face of adversity. With TikTok they were able to share activities, create a platform for their own creative expression, and most of all through their own agentive actions develop forms of digital play.	1) Lack of validated questionnaire 2) Small sample size	5/9
Feijoo et al., [80]	Cx	1055 (46%)	11–17	Spain	Self-developed questionnaire; the Silhouette Test.	Ideal- and self-body image, Satisfaction of physical appearance.	Exposure to advertising by influencers on SM such as YouTube, Instagram, and TikTok does not influence adolescents’ perception of their own bodies, but it is directly related to lower body satisfaction and their perceived importance of physical appearance to others, both to friends and to people with whom they have a more distant or no relationship.	1) Lack of SM use evaluation	5/9
Fortunato et al., [67]	Cx	340 (38%)	13–19	Italy	Self-developed questionnaire; the BMSAS; the INCOM; the DERS; the Young Person’s CORE.	SM addiction, Emotional regulation, and Psychological distress.	Regarding TikTok, the results of a three-class model showed that heavy users reported levels of addictive SM use, online social comparison, and emotion dysregulation close to those of low users. The other class, the lowest users, had the lowest scores for all the indicators.	1) Non-objective measures of smartphone use 2) Lack of validated questionnaire	4/9
Gentzler et al., [71]	L	237 (51%)	14–16	US	Self-developed questionnaire; the Rosenberg self-esteem scale; the BFI‐2‐XS; a self-developed scale to investigate the negative affective reactions to social media; CDI‐2.	Self-esteem, Personality traits, Negative affective reactions to social media, and Depressive symptoms.	Less extroverted teens and teens prone to negative reactions to SM reported elevated depressive symptoms when using SM more, particularly Instagram and TikTok.	1) Lack of validated questionnaire 2) Influence of the COVID-19 outbreak 3) Low diversity in the population	4/9
Hull et al., [76]	CS	6 (100%)	13–16	US (TX)	Description of clinical cases.	Tic-like movements.	Each patient reported watching videos of tics on TikTok made by the same influencer before the onset. Most common tics in the cohort are also seen in the influencer.	1) Small sample group 2) Lack of validate assessment 3) Selection bias	5/9
Ilic-Zivojinovic et al.,[68]	Cx	620 (67%)	14–19	Serbia	Self-developed questionnaire; the IAT; the CES-DC.	Internet addiction and Depressive symptoms.	The use of TikTok was significantly more frequent among internet addicts compared to internet normal users and is an independent predictor of a high total score at the CES-DC.	1) Conducted during the COVID-19 outbreak 2) Lack of potentially relevant sociodemographic and health informations	5/9
López-Gil et al., [81]	Cx	653 (56%)	12–17	Spain	Self-developed questionnaire; the SNAddS-6 S; the SCOFF questionnaire.	Social Media addiction and Eating Disorders).	No significant correlation emerged between TikTok and ED assessed by SCOFF (bivariate correlation). Adolescents with high SM addictive behaviors showed a higher likelihood of presenting ED.	1) Desirability bias 2) Recall bias 3) Non-validated questionnaire used for ED	4/9
Maes et al., [73]	L	229 (100%)	10–19	Belgium	Self-developed questionnaire; the SATAQ-4; the 9-figure contour scale.	Internalization of beauty ideals and body image of self-discrepancy.	TikTok was not predictive of subsequent increases or decreases in girls’ internalization of beauty ideals and body image self-discrepancy over time. Potentially only contribute to girls’ body image constructs in the short term. Autoregressive paths were significant for TikTok, meaning that for girls who experience an increase in their personal level of TikTok use at a certain point in time, this level will further increase at a subsequent time point.	1) High rate of drop-out 2) Geographic limitation	6/9
Marengo et al., [69]	Cx	765 (52%)	11–19	Italy	Self-developed questionnaire; the BSMAS.	SM addiction	The time spent on smartphones was the strongest predictor of SM addiction, followed by TikTok use. Adolescents reporting use of TikTok in combination with other high-visual SM platforms typically showed higher risk of SM addiction than adolescents not using TikTok.	1) Self-reported survey 2) Studied only lockdown period	5/9
Muñoz- Rodríguez et al., [82]	Cx	1991 (57%)	12–18	Spain	Self-developed questionnaire; the CHAID algorithm decision tree.	Isolation, Unhappiness, Dissatisfaction, Abuse, Bullying, and Time management	Young people that do not feel isolated when not online and make limited or no use of videogames and TikTok affirm that they experience a sense of risk regarding their online use.	1) High risk of obsolescence 2) Selection bias 3) A specific data mining method cannot be confirmed	4/9
Nagy et al., [77]	CS	5 (100%)	10–18	Hungary	Description of clinical cases.	Functional tics.	TikTok may act as a trigger of the abruption of functional tics. The presentation of tics by SM influencers, especially during a period of scarcity of the normal in-person social stimuli, may lead to the development or exacerbation of tics (or tic-like movements) in children watching these videos.	1) Small sample group 2) Lack of validated assessment 3) Selection bias	5/9
Pruccoli et ali, [42]	Cx	78 (94%)	10–19	Italy	Self-developed questionnaire.	Self-esteem.	Patients who reported longer periods spent each day using TikTok, more frequently described a negative effect on their self-esteem. Patients reporting changes in their daily lives due to TikTok described a higher frequency of searches for pro-ED recovery contents.	1) Selection bias 2) Lack of validated questionnaire	5/9
Qin et al., [84]	Cx	633 (49%)	10–19	China	Self-developed questionnaire.	Enjoyment, Concentration, Time Distortion, Active Parental Mediation, and Problematic TikTok Use.	As part of the flow experience, concentration and time distortion were positively associated with adolescents’ problematic TikTok use. Enjoyment, the first stage of flow, is not associated with the problematic use of TikTok. Active parental mediation negatively moderated the relationship between concentration and problematic TikTok use.	1) Purposive sampling 2) Limited other psychosocial variables considered 3) Geographic limitation	5/9
Qin et al., [83]	Cx	659 (56%)	10–19	China	Self-developed questionnaire.	Enjoyment, Concentration, Time distortion, and TikTok addiction behavior.	Information quality (e.g. conciseness, usefulness) and system quality (e.g. flexibility, integration, ease of use, response time) have a partial positive influence on flow experience and a positive effect on TikTok addiction behavior. System quality contributed more to users’ flow experience and addiction behavior than information quality. Among the three factors contributing to the flow experience (enjoyment, concentration, time distortion), concentration was the most important factor in TikTok addiction behavior.	1) Geographic limitation 2) Focus on one short-video application	5/9
Sagrera et al., [70]	Cx	5070 (54%)	14–19	US (LA)	Self-developed interview.	Self-reported body-image issues.	TikTok shows a statistically significant difference in self-reporting Body-image issues (BII). It also shows the second higher odds of self-reporting BII. When stratified by sex, predictors (SM used, time spent on SM daily, number of SM used), shows increased odds of reporting BII in both females and males who use TikTok.	1) Selection bias 2) Lack of validated questionnaire 3) Conducted before the COVID-19 outbreak	5/9
Sarman et al., [72]	Cx	1176 (58%)	13–18	Turkey	Self-developed questionnaire; the UCLA Loneliness Scale; the Adolescent Anger Rating Scale.	Loneliness, Anger, and SM Attitude.	TikTok use was not related to loneliness scores, but the mean scores of anger-reactive, anger-instrumental, and anger-total scores of these users were statistically higher. The duration of TikTok usage was not related to loneliness and anger scores.	1) Geographic limitations 2) Lack of information about the participants’ personality traits or the emotions experienced while using SM	6/9
Sha et al., [85]	Cx	3036 (57%)	14–18	China	Smartphone Addiction Scale short version adapted for TikTok; Depression Anxiety Stress Scales 21; forward and backward digit spans.	Depression, Stress, Anxiety, and Working memory capacity.	TikTok use disorder is positively linked to memory loss and to depression, anxiety, and stress. Depression, anxiety, and stress are positively linked to memory loss. Depression, anxiety, and stress have a mediating effect between TikTok use disorder and memory loss.	1) Geographic limitation	5/9
Soriano- Ayala et al., [78]	Cx	12 (50%)	8–17	Spain	Study 1: adapted and abbreviated version of the scientific scale of Graff et al. (2013).	Sexualized behaviors.	Study 1: most observed sexualized behaviors were present in the videos of both female and male TikTokers without statistically significant differences.	1) Lack of validated questionnaire 2) Small sample size	4/9
					Study 2: self-developed semi-structured interview; thematic analysis of contents.	Self-perception and Mental health.	Study 2: minors openly recognize sexualization in the videos as a characteristic feature of TikTok. Hypersexualization is assumed to be a strategy to improve the virtual self that contributes to capturing attention, accumulating recognition in the form of likes or followers, monopolizing social prestige, and even monetizing the capitalization of accumulated influence.		
Wu et al., [79]	Cx	659 (60%)	3–17	China	Self-developed questionnaire; the Satisfaction with Life scale; the Positive Affect and Negative Affect Scale for Children.	Life satisfaction and Positive and Negative affects.	Females displayed more active use of social short-form video platforms. Watching more Entertainment/Relaxation videos is associated with higher life satisfaction, whereas watching People/Fashion videos is associated with lower life satisfaction. Passive use of social short-form videos predicted reduced life satisfaction and positive affect, whereas active use, particularly posting videos, predicted enhanced life satisfaction.	1) Geographic limitation 2) Small effect-size	6/9

Cx: cross-sectional studies. L: longitudinal studies. CS: case series studies. SM: Social Media. ED: Eating Disorder. CHAID: Chi-square Automatic Interaction Detector. BMSAS: Bergen Social Media Addiction Scale. INCOM: IOWA-Netherlands Comparison Orientation Measure. DERS: Difficulties in Emotion Regulation Scale. BFI-2-XS: extra-short form of the Big Five Inventory 2. CDI-2: Children’s Depression Inventory 2. IAT: Internet Addiction Test. CES-DC: Center for Epidemiological Studies Depression Scale for Children. SNAddS-6 S: Short Social Networks Addiction Scale-6 Symptoms. SCOFF: Sick, Control, One, Fat, Food questionnaire. SATAQ-4: Sociocultural Attitudes Towards Appearance Questionnaire-4. See Table S3 (online resources) for references of the assessment scales included

### General characteristics of the studies

All included studies were published between 2020 and 2023. Quality index of the retrieved studies ranged between 4 and 6 points out of 9, with an average of 4.8 and a median score of 5, which corresponds to “fair quality” according to the Agency for Healthcare Research and Quality (AHRQ) standards [66]. Fifteen studies used a cross-sectional design, two used a longitudinal design and three studies applied non-quantitative methods (two case series, and one narrative inquiry). None of the studies included control groups. Sample sizes of studied groups varied between 5 and 5070, with minimum age of 2 and a maximum of 19 years. Globally, a total of 17,312 subjects from ten countries were evaluated. Europe was the most represented continent, with eleven studies conducted with adolescent populations from Italy, Spain, Denmark, Hungary, Serbia, and Belgium. The other studies were conducted in China (4 studies), the US (3 studies), Turkey (1 study) and Canada (1 study). Socio-economic status was investigated in five articles only, though unsystematically. We grouped and synthesized the selected studies based on the findings related to the characteristics of the SM analyzed, the tests/measures used, and the topic of interest (Table 1).

### Characteristics of SM use and mental health evaluation

Among the included studies, only four reported data about total daily screen time [67–70], and four detailed the amount the time spent on TikTok [42, 67, 71, 72]. Specifically, Pruccoli et al. reported that for 62.8% of the analyzed sample TikTok represented the preferred SM platform and was used for a mean of 1.4 ± 1.0 h/day [42]. One study [73] did not report the exact time spent on TikTok but referred to the stability of use over a 9-month period. TikTok can engage users in various ways: actively through creating and sharing their own content or by interacting and commenting on others’ content, or passively by simply scrolling through videos. Given that, ten studies differentiated the population between active and passive users [42, 67, 70, 72, 74–79], while only one conducted a content analysis of the posts, hashtags, or videos [78]. Four of the studies [68, 75, 79, 80] assessed wellbeing indicators, like creativity, innovation, life satisfaction, and health habits, while sixteen assessed mental disorder indicators such as depression, anxiety, stress or body image issues [42, 67, 68, 70–73, 76–79, 81–85]. Eleven studies used self-developed questionnaires for outcome evaluation [42, 67–72, 74, 80–82], of which only six paired it with standardized measures [67–69, 71, 72, 80, 81]. Six studies used only standardized tests (in their original or adapted form) [73, 78, 79, 83–85] while three studies did not administer any questionnaire but conducted unstructured interviews with the subjects or a descriptive analysis [75–77].

### Main focus of included studies

#### TikTok and mental health

Seven studies tried to evaluate the relationship between TikTok use and its effects on mental health. *Burke et al.* [75] evaluated the possible positive implications of SM use during the COVID-19 pandemic. They found that with the use of TikTok, children were more able to share activities, create a platform for their creative expression, and engage in new and alternative forms of digital play. Analyzing the negative impact of TikTok on mental health, *Wu et al.* [79] showed that a greater number of hours spent on TikTok (passive use) predicted lower life satisfaction and had an inverse association with positive emotions while the number of videos posted (active use) predicted higher life satisfaction. *Sarman et al.* [72], on the other hand, studied the relationship of TikTok with loneliness and anger levels of adolescents. They found that the duration of TikTok use was not related to loneliness and anger, but the mean anger scores were statistically higher in those who used TikTok compared to other SM users. *Ilic-Zivojinovic et al.* [68]. showed that TikTok use is significantly more frequent among internet addicts, and the time spent on this platform is highly associated with more depressive symptoms. *Gentzler et al.* [71] highlighted that less extroverted teens and those most prone to negative reactions reported elevated depressive symptoms when using TikTok. Finally, two studies [76, 77] analyzed the possible impact of TikTok videos on the spread of functional motor disorders or tic-like movements. The studies suggested that SM may contribute to the spread of functional neurological symptom disorder, especially during a period of less in-person social interactions (COVID-19 pandemic).

#### Problematic TikTok use

Problematic TikTok use (PTU) is defined as the uncontrolled and obsessive use of TikTok, which may have negative physical or psychosocial consequences [84, 86]. However, in literature, the term is used more broadly and indefinitely, often as a synonym of TikTok addiction or use disorder [87]. Seven studies evaluated PTU analyzing both its causes and risks. *Marengo et al.* [69] found that time spent on the smartphone and TikTok were the strongest predictors of risk of SM addiction. In two subsequent studies, *Qin et al.* [83, 84] explored the role of the “flow experience” as a mediator for PTU. They first found that TikTok’s intrinsic characteristics facilitated the flow experience by inducing higher “concentration” on the platform’s content and distorted time perception [83]. These aspects were later linked to the onset of PTU, while parental control emerged as a protective factor in the correlation between concentration issues and PTU [84]. *Sha et al.* [85] showed that TikTok use disorder was positively linked to memory loss, depression, anxiety, and stress. *Fortunato et al.* [67] divided their sample into groups based on time spent on TikTok and matched them with psychosocial characteristics such as social comparison, difficulties in emotional regulation, and psychological distress, revealing that, despite significant differences in time spent on the app (2 h vs. 9 h), groups did not differ in psychosocial characteristics. Also, *Muñoz-Rodríguez et al.* [82] highlighted that males with a greater sense of isolation when offline and greater TikTok use were more frequently exposed to online risks, such as cyberbullying, excessive use, inappropriate behavior, and identity theft. *Bucknell Bossen et al.* [74], in their study, described three different types of TikTok users (passive, participatory, and contributory) and tried to define motivations underlying use patterns in pre-adolescents and adolescents. According to their study, passive use was mostly associated with affective needs, with no differences across variable gender, age, ethnic, or intensity of use. Entertainment and emotional arousal served as primary drivers for all types of users, but “fame-seeking” behaviors more clearly emerged as a source of gratification for the heavy contributory users, particularly among pre-adolescent girls.

#### TikTok, body image, and self-esteem

Six studies analyzed the impact of TikTok use on the construction of body-image ideals and self-esteem. *Sagrera et al.* [70] showed that TikTok users had a high probability of self-reporting body-image issues. *Feijoo et al.* [80]. showed that exposure to advertising on TikTok was directly related to lower body satisfaction and higher perceived importance of physical appearance to others. Similarly, in the work of *Pruccoli et al.* [42], patients spending more time per day on the app reported lower self-esteem levels both secondary to passive use and the search for content about food, diet, or eating disorders. *Maes et al.* [73], on the other hand, demonstrated that time spent on TikTok was not influential on perceived body image, and beauty ideals. *Lòpez-Gil et al.* [81]. also found no significant correlation between TikTok and the presence of eating disorders. *Soriano-Ayala et al.* [78] explored the phenomenon of hypersexualization on TikTok. The authors interpreted the hypersexualized self-representations, on one hand, as a strategy to improve self-efficacy according to standards of beauty, and popularity, while on the other, as potential source of peer criticism and pressure, significantly impacting self-esteem.

## Discussion

This systematic review presents several insights into the relationship between TikTok usage and different aspects of mental health in youth. Notably, all the studies included in the review were published within the past four years, indicating a considerable research focus on the topic. However, despite the growing interest in this area, the available literature reveals a deficiency in targeted and high-quality studies within this demographic.

The subject of screen media use and neurodevelopment has become a surprisingly contentious issue. Several studies have highlighted that increased caregiver screen use in early childhood and exposure to age-inappropriate content, such as violent or mature-audience programming, is associated with poorer cognitive and psychosocial outcomes [88]. Conversely, the co-use of screen media between caregivers and children, better-quality content, such as educational programming, and later screen exposure during childhood have been positively associated with cognitive and language development [88, 89].

Thus, context of use beyond screen time has been gaining increasing consideration in the debate around the effects of screen media use on children and adolescents. Further, with regards to mental health outcomes, even if a recent meta-analysis points to a correlation between screen time and both internalizing and externalizing behavior problems in children under 12 years, findings reveal that effect sizes of most analyzed studies are small [90], indicating that screen use might be just one contributor among many factors embedded within multiple organizational layers [56, 88].

In the present study, we focused on the impact of a specific SM platform (TikTok), during a definite age range (adolescence), related to a determinate outcome (mental health). Except for the study by *Burke et al.* [75], most included articles indicated an overall negative impact of TikTok on the mental health of adolescent users. The analyzed effects encompassed lower life satisfaction [79], increased depressive symptoms [68], higher levels of anger and loneliness [72], risk of PTU [69, 83, 84], increased body image concerns, and self-esteem issues [42, 70, 80], as well as the rapid spreading of functional tic-like behaviors [76, 77]. Prior research on population data found that the relationship between SM use and adolescents’ life satisfaction is not univocal, with changes across different developmental ages and according to sex [91]. Higher SM use has been highlighted as a predictor of decreased life satisfaction in females aged 11–13 and males aged 14–15 years particularly, which suggests the presence in teens of different windows of sensitivity to SM [91]. This observation likely reflects the higher interpersonal sensitivity and social comparison tendencies during the younger adolescence period. However, if adolescence-specific pubertal, cognitive, and social changes may undoubtedly underlie the developmental windows of sensitivity, variations in individual motivations and personality traits may be also relevant in shaping the effects of TikTok use on adolescents’ mental health. The search for gratification through entertainment or new potential social connections appears as one of the primary drivers for increased SM use in teens [74]. Further evidence suggests that being an active user (e.g., posting videos on SM) may be even more directly associated with the proneness to spend greater time on SM independently from the amount of received “likes,” a commonly used metric of gratification [79]. Thus, the need to receive a concrete “gain” through SM consumption, whether resulting from social connectedness, entertainment or personal expression by content production, appears as a potential mediator of increased TikTok use and its possible mental health implications. An interesting conceptual framework in this regard is the mental effort-gratification model [59], according to which adolescents may have different levels of gratification and investment while using SM according to four user categories. The model encompasses two dichotomous user profiles: the ‘lucky ones’, who experience the most favorable outcomes despite little mental effort in posting or interacting with others on SM, and the ‘sufferers,’ who, despite high investment in SM use, receive low gratification. Adolescents fitting into the sufferer category are probably more susceptible to social acceptance and may represent an “endophenotype” at risk for experiencing more adverse mental health outcomes. There are seemingly different individual factors that may place users at different susceptibility risk towards SM, including the presence of psychological problems and specific personality traits. Higher social anxiety and social comparison tendencies, as well as inadequate emotion regulation strategies, may play a crucial role in this regard [67]. Further, introverted teens who use TikTok and show more negative emotional arousal to its contents, are at risk of developing higher depressive symptoms after one year of use [71]. Taken together, these findings highlight that time spent on TikTok and SM is probably less informative per se in terms of mental health, whereas differences in age, sex, personality, motivations behind, and gains deriving from self-exposure may be more relevant informants in the exploration of the complex relationship between TikTok and wellbeing in youth.

A relevant part of the included studies focused on the risk of developing addictive TikTok use behavior or PTU. Some of them highlighted that time spent on TikTok correlates both with PTU as well as risk of mental distress and depressive symptoms in teens [68, 69, 85]. Conversely, other studies showed that the variable “time” is not sufficiently sensitive to predict PTU risk as it does not account for individual factors (e.g., emotion regulation abilities) involved in dysfunctional TikTok usage patterns [67]. A parallel line of research has pinpointed that TikTok’s specific features may be more directly linked to this outcome. TikTok’s system quality elements (flexibility, integration, ease of use, and response time) are particularly designed to foster intense concentration and immersion into loops of contents, the so-called “flow experience”. This immersive state has been identified as a significant factor influencing addictive behaviors related to TikTok [83] as it can lead to a disregard for the immediate surroundings and induce time distortion, both of which correlate with PTU severity [84]. Additional features of TikTok may further explain the risk of inducing addictive behaviors. For instance, TikTok has a strong algorithm-driven presentation of contents compared to other SM, with content consumption forming the primary experience on the platform. This distinguishes TikTok from other popular SM, where algorithms are more frequently used to facilitate user interactions rather than primarily shaping content consumption [92]. Consequently, TikTok’s algorithmic prominence results in the creation of a personalized “algorithmic version” of each user, categorized according to predefined schemas [93–95]. This phenomenon bears not only the potential to reshape traditional models of self-presentation on SM but may also contribute to PTU by strengthening users’ attachment to the platform. This hypothesis still requires further investigation in future studies and, overall, the available evidence on TikTok and risk of PTU is mixed. This is likely due to the absence of a clear definition of what “problematic” use is, warranting further research on the long-term effects of sustained use on adolescents, particularly in terms of possible modulation of their cognitive and social abilities. Specific attention should be also devoted to the interaction between individual psycho(patho)logical factors and TikTok’s unique features.

TikTok’s specific characteristics have also been associated with consequences on body image, self-perception, and self-esteem in adolescents [96]. Bodies are consistently showcased across the platform [96] and since its first appearance, TikTok has been encouraging the sharing of body-centered content (e.g., dance challenges). These features may enhance/intersect typical developmental and social processes in adolescence, which include the increased salience of peer feedback [97, 98] and the heightened self-consciousness in the form of the “imaginary audience” [99]. This makes TikTok particularly valid as an online stage whereby adolescents shape their identity and estimate their social value through the exhibition of their own bodies. However, the platform may also create a challenging environment for the development of self-acceptance given the massive presence of content encouraging weight loss, and videos and images frequently edited according to idealized standards of beauty, often in ways adolescents are unable to detect [100]. In female teens, the presence of body image concerns may be a key mechanism leading from SM use to negative mental health outcomes by amplifying the importance placed on physical appearance and increasing the focus on receiving approval through measurable indicators [98]. While time spent on TikTok has not been clearly linked to an higher likelihood of eating disorders and body dissatisfaction [73, 81], most of the review’s studies reported negative effects of food-, diet- or weight-related contents on body image [42, 71], increasing in users the perceived importance of their physical appearance to others [80]. Adolescents not only expose their bodies on TikTok but often sexualize their outfits and behaviors as a means to gain greater recognition and attention from their peers in the form of “likes” and followers. Although some adolescents perceive self-sexualization as a form of empowerment, posting sexualized body content may also expose them to significant criticism and peer pressure [78], potentially increasing body concerns. These findings underscore the intricate interplay between TikTok, body image perceptions, and mental health outcomes. On teens’ developing sense of identity and validity, body-centered contents hold the risk of encouraging unrealistic body ideals. Addressing these complexities is crucial to promote a healthier online environment and a culture of diversity and self-acceptance.

SM offer their users the opportunity of unprecedented and extraordinary visibility. As such, SM hold the potential capacity to influence many human behaviors, both safe or unsafe, healthy or unhealthy. For instance, extensive research has been conducted on the influence of SM on violent behavior, with strong evidence that real-life violence in youth can be modeled by mass media influence [101, 102]. Similarly, the SM-based exposure to auto-aggressive behaviors or other illness behaviors may represent a possible source of “contagion” of such symptoms among teens [31, 103]. TikTok has lately received increasing research scrutiny as a potential ‘spread vector’ for mental illness symptoms and disorders [29, 104]. This is due to TikTok’s emergence as a privileged environment for teens to showcase mental health distress or promote peer support, with nearly half of the platform’s most viewed content featuring mental health hashtags or referring to symptoms of poor mental health [44]. Since 2019, there has been a well-documented inflation of content creators who described having tics or Tourette syndrome on TikTok [30, 105], which coincided with massive presentations of adolescents to tertiary tic clinics, principally in Germany and Northern America [30, 106]. Accordingly, two studies of this review documented that presentation of tic-like behaviors by popular influencers on TikTok, especially during a period of scarce in-person interactions, has acted as trigger for the abruption of functional tics in teens [76, 77]. Similar dynamics of SM modeling of peri-psychiatric symptoms in youth have consistently been reported with respect to dissociative identity disorder [107, 108] and self-harm [109–111]. The ongoing evolution of this trend emphasizes the pressing need for a deeper understanding of the impact of SM on mental health. This includes exploring the contemporary clinical manifestations of mental distress in youth and considering the potential for algorithm-driven SM platforms to facilitate the spread of self-diagnosed mental health conditions in most vulnerable teens through mechanisms of social contagion.

## Limitations and future directions

The main limitation of the reviewed studies is inherent to their cross-sectional methodology and the use of solely correlational data, which hampers the understanding of causal relationships between TikTok use and mental health outcomes. At the same time, even in the eventual availability of longitudinal population-based data from countries with different socioeconomic conditions, we might still lack sources of interpretation of the current mental health crisis among contemporary adolescents. This may be due to the probably over-reductionist approach in understanding mental health as the result of a single factor.

In particular, several potential biases need to be considered when interpreting the findings of the studies included in this systematic review. Selection bias is a common issue, as many studies used non-randomized samples, often relying on convenience sampling or self-selection. This approach may limit the generalizability of the results since the study populations - despite including approximately 17,000 participants across all retrieved studies - might not accurately represent the broader adolescent TikTok user base. To mitigate this in future research, employing randomized sampling methods or stratified sampling techniques that better reflect the demographic characteristics of the adolescent population would be beneficial.

Reporting bias also poses a challenge, particularly in studies that depend on self-reported data for TikTok usage and non-standardized mental health evaluations. Self-report measures can be influenced by participants’ perceptions, memory recall, and willingness to disclose, leading to inaccuracies. The absence of standardized assessment tools across studies contributes also to measurement bias, making it difficult to compare results and draw firm conclusions about TikTok’s impact on adolescent mental health. Even when validated tests to track SM use were employed, these measures may fail to adapt to the continuously evolving trends and software updates typical of all SM. Future studies should consider incorporating objective measures of TikTok use, such as app usage data, alongside validated psychometric tools to assess mental health outcomes, facilitating cross-study comparisons and enhancing the reliability of findings.

Publication bias might also have skewed the existing evidence, as studies with significant findings are more likely to be published, potentially overshadowing other results. This bias could lead to a disproportionate focus on the adverse effects of SM, including TikTok, while potentially underreporting or neglecting the positive aspects associated with these platforms. The predominance of research highlighting negative outcomes found in our review might have been the result of this bias and may lead to an incomplete understanding of TikTok’s overall impact. To address this, future systematic reviews might include comprehensive search strategies that encompass gray literature and unpublished studies, ensuring a more balanced view of the evidence; however, it is evident that more rigorous research on this topic is needed to explore how user-specific psychopathological characteristics may interact with TikTok’s peculiar features.

## Conclusions

The relationship between social media (SM) and mental health is intricate, and research in this area is still in its early stages. Although TikTok is particularly popular among adolescents, its effects on well-being are complex and multifaceted. While there is widespread concern and correlational evidence pointing to potential negative impacts, it is crucial to approach this topic with caution. The tendency of authorities to issue warnings and recommendations may create the impression of a scientific consensus on the detrimental effects of SM on youth, justifying the imposition of bans and restrictions. However, as Odgers [112] rightly notes, there is no definitive evidence that SM, including TikTok, is “rewiring children’s brains” or driving a widespread epidemic of mental health disorders. This observation is also supported by the inconclusive evidence from our systematic review.

Nevertheless, at least a subset of adolescents appears particularly vulnerable to developing psychiatric symptoms following exposure to representations of mental illness on SM. This suggests that while TikTok and similar platforms may pose risks, these risks are likely mediated by individual factors, such as pre-existing mental health conditions and specific personality traits. Therefore, a blanket stigmatization of TikTok is unwarranted. The mechanisms of pathological spread, which have long existed in psychiatry, operate through diverse “routes” and are shaped by evolving and highly influential social interactions [113].

As the landscape of child and adolescent mental health evolves alongside the digital age, clinicians must adapt their approaches to address the challenges posed by SM. By integrating structured SM anamnesis, understanding the nuances of SM use, and fostering open, non-judgmental communication with young patients, clinicians can better navigate the dual-edged nature of digital connectivity. While the surge in mental health issues among young individuals parallels the widespread adoption of SM, it is essential to recognize that SM is not inherently detrimental. Instead, SM platforms mirror the complexities of modern adolescence, offering both risks and opportunities.

A proactive, holistic approach enables clinicians to harness the positive potential of SM while safeguarding the mental well-being of young individuals. By adopting a comprehensive and developmentally grounded perspective, we can better understand the complex interplay between TikTok and adolescent mental health. This understanding will be critical in informing policymakers, educators, parents, and mental health professionals, enabling them to develop evidence-based interventions and policies that balance the potential risks and benefits of TikTok - and other SM - use among adolescents.

## Electronic supplementary material

Below is the link to the electronic supplementary material.

Supplementary Material 1

Supplementary Material 2

## Data Availability

Data availability: Data will be made available upon request.
