# Supplementary material for: Scrolling through adolescence: a systematic review of the impact of TikTok on adolescent mental health
Source: Eur Child Adolesc Psychiatry. 2024 Oct 16;34(5):1511–27. doi: 10.1007/s00787-024-02581-w (PMC12122552; doi:10.1007/s00787-024-02581-w)
Supplement: Supplementary file 1 — Supplementary Material 1 [file 787_2024_2581_MOESM1_ESM.docx]

**Tab. 1S**

Adapted version of the Newcastle-Ottawa scale used in the present study for quality assessment (maximum 9 stars).

| **NEWCASTLE - OTTAWA QUALITY ASSESSMENT SCALE**  **Selection** (max 4)  1) Representativeness of the group exposed to TikTok  a) truly representative of the average child or adolescent in the community **✵**  b) somewhat representative of the average child or adolescent in the community **✵**  c) selected group of users (e.g. volunteers, influencers)  d) no description of the derivation of the cohort  2) Selection of the group non exposed to TikTok  a) drawn from the same community as the exposed group**✵**  b) drawn from a different source  c) no description of the derivation or absence of a non-exposed group  3) Ascertainment of exposure  a) secure record (e.g. direct observation, screenshots) **✵**  b) interview or specific questionnaire about TikTok use **✵**  d) no description (e.g. not clear if the child/adolescent has been directly exposed to TikTok)  4) Demonstration that outcome of interest was not present at start of study  a) yes **✵**  b) no  **Comparability** (max 2)  1) Comparability of groups on the basis of the design or analysis  a) study controls for age and gender **✵**  b) study controls for type of use (active vs passive use) **✵**  c) no description  **Outcome** (max 3)  1) Definition of outcome   1. clear definition of the outcome of interest **✵** 2. no description   2) Assessment of outcome  a) standardized interviews or questionnaires **✵**  b) record linkage **✵**  c) non-standardized measures  d) no description  3) Drop-outs rate:  a) No drop-outs or same rate for all groups. **✵**  b) Different rate or not defined  c) No description or absence of more than one group |
| --- |

**Tab. 2S**

Details on quality assessment indices for the retrieved studies.

|  | **Study** | **Selection (max 4*)** | | | | **Comparability (max 2*)** | **Outcome (max 3*)** | | | **Total (max 9*)** |
| --- | --- | --- | --- | --- | --- | --- | --- | --- | --- | --- |
|  |  | **Representativeness of the group exposed to TikTok** | **Selection of the group non exposed to TikTok** | **Ascertainment of exposure** | **Demonstration that outcome of interest was not present at start of study** | **Comparability of group on the basis of the design or analysis** | **Definition of outcome** | **Assessment of outcome** | **Drop-outs rate** |  |
| **1** | Bucknell Bossen et al, 2020 | *(a) | (c) | *(b) | (b) | *(a) | *(a) | (c) | (c) | **4** |
| **2** | Burke et al, 2023 | *(a) | (c) | *(b) | (b) | ** (a)(b) | *(a) | (c) | (c) | **5** |
| **3** | Feijoo et al, 2023 | *(a) | (c) | *(b) | (b) | *(a) | *(a) | *(a) | (c) | **5** |
| **4** | Fortunato et al, 2023 | *(a) | (c) | *(b) | (b) | (c) | *(a) | *(a) | (c) | **4** |
| **5** | Gentzler et al, 2023 | *(a) | (c) | *(b) | (b) | (c) | *(a) | *(a) | (c) | **4** |
| **6** | Hull et al, 2021 | *(a) | (c) | *(b) | (b) | ** (a)(b) | *(a) | (c) | (c) | **5** |
| **7** | Ilic-Zivojinovic et al, 2023 | *(a) | (c) | *(b) | (b) | *(a) | *(a) | *(a) | (c) | **5** |
| **8** | López-Gil et al, 2023 | *(a) | (c) | *(b) | (b) | (c) | *(a) | *(a) | (c) | **4** |
| **9** | Maes et al, 2022 | *(b) | (c) | *(b) | *(a) | (c) | *(a) | *(a) | (b) | **5** |
| **10** | Marengo et al, 2022 | *(a) | *(a) | *(b) | (b) | (c) | *(a) | *(a) | (c) | **5** |
| **11** | Muñoz-Rodríguez et al, 2023 | * (a) | (c) | *(b) | (b) | *(a) | *(a) | (c) | (c) | **4** |
| **12** | Nagy et al, 2022 | *(a) | (c) | *(b) | (b) | ** (a)(b) | *(a) | (c) | (c) | **5** |
| **13** | Pruccol et ali, 2022 | * (a) | (c) | *(b) | (b) | ** (a)(b) | *(a) | (c) | (c) | **5** |
| **14** | [Qin](https://doi.org/10.3389/fpsyg.2022.932805) et al, 2022 | *(a) | (c) | *(b) | (b) | *(a) | *(a) | *(a) | (c) | **5** |
| **15** | [Qin](https://doi.org/10.3390/ijerph20032089) et al, 2023 | *(a) | (c) | *(b) | (b) | *(a) | *(a) | *(a) | (c) | **5** |
| **16** | Sagrera et al, 2022 | * (a) | (c) | * (b) | (b) | ** (a)(b) | * (a) | (c) | (c) | **5** |
| **17** | Sarman et al, 2023 | *(a) | (c) | *(b) | (b) | **(a)(b) | *(a) | *(a) | (c) | **6** |
| **18** | Sha et al, 2021 | * (b) | (c) | *(b) | (b) | * (a) | * (a) | *(a) | (c) | **5** |
| **19** | Soriano-Ayala et al, 2022 | (c) | (c) | **(a)(b) | (b) | (c) | *(a) | *(a) | (c) | **4** |
| **20** | Wu et al, 2021 | *(a) | C | *(b) | *(a) | *(a) | *(a) | *(a) | (c) | **6** |
|  | ***Note:* the specific fulfilled criterion for the assignment or non-assignment of each star is specified in brackets.** | | | | | | | | | |

**JOURNAL:** [**European Child & Adolescent Psychiatry**](https://link.springer.com/journal/787)

**ARTICLE TITLE: Scrolling through Adolescence: a systematic review of the Impact of TikTok on adolescent mental health**

Giulia Conte^1a^, Giorgia Di Iorio^1a^, Dario Esposito^1^, Sara Romano^1^, Fabiola Panvino^1^, Susanna Maggi^1^, Benedetta Altomonte^1^, Maria Pia Casini ^1^, Mauro Ferrara^1,^, Arianna Terrinoni^1*^

^a^ these authors contributed equally to this work

^1^ *Department of Human Neuroscience, Unit of Child and Adolescent Neuropsychiatry, Sapienza University of Rome, Via dei Sabelli 108, 00185 Rome, Italy*

**Corresponding author***:*

Arianna Terrinoni, MD

E-mail: a.terrinoni@policlinicoumberto1.it
