# Supplementary material for: Scrolling through adolescence: a systematic review of the impact of TikTok on adolescent mental health
Source: Eur Child Adolesc Psychiatry. 2024 Oct 16;34(5):1511–27. doi: 10.1007/s00787-024-02581-w (PMC12122552; doi:10.1007/s00787-024-02581-w)
Supplement: Supplementary file 2 — Supplementary Material 2 [file 787_2024_2581_MOESM2_ESM.docx]

**Tab. 3S**

**BIBLIOGRAPHIC REFERENCES OF THE TOOLS USED IN THE ARTICLES INCLUDED IN THE REVIEW**

| **Studies** | **Assessment Tools** | **References** |
| --- | --- | --- |
| Bucknell Bossexn et al, 2020 | Self-developed questionnaire. | *none* |
| Burke et al, 2023 | Day-in-the-life methodology and narrative inquiry. | *none* |
| Feijoo et al, 2023 | Self-developed questionnaire; the Silhouette Test. | - Thompson, M. A., & Gray, J. J. (1995). Development and Validation of a New Body-Image Assessment Scale. *Journal of Personality Assessment*, *64*(2), 258–269. |
| Fortunato et al, 2023 | Self-developed questionnaire; the BMSAS; the INCOM; the DERS; the Young Person’s CORE. | - Schou Andreassen, C., Billieux, J., Griffiths, M. D., Kuss, D. J., Demetrovics, Z., Mazzoni, E., & Pallesen, S. (2016). The relationship between addictive use of social media and video games and symptoms of psychiatric disorders: A large-scale cross-sectional study. *Psychology of Addictive Behaviors: Journal of the Society of Psychologists in Addictive Behaviors*, *30*(2), 252–262. - Gibbons, F. X., & Buunk, B. P. (1999). Individual differences in social comparison: Development of a scale of social comparison orientation. *Journal of Personality and Social Psychology*, *76*(1), 129–142. - Gratz, K. L., & Roemer, L. (2003). Multidimensional Assessment of Emotion Regulation and Dysregulation: Development, Factor Structure, and Initial Validation of the Difficulties in Emotion Regulation Scale. *Journal of Psychopathology and Behavioral Assessment*. - Twigg, E., Barkham, M., Bewick, B., Mulhern, Connell, J., & Cooper, M. (2009). The Young Person’s CORE: Development of a brief outcome measure for young people. *Counselling and Psychotherapy Research*, *9*, 160–168. |
| Gentzler et al, 2023 | Self-developed questionnaire; the Rosenberg self‐esteem scale; the BFI‐2‐XS; a self-developed scale to investigate the negative affective reactions to social media; CDI‐2. | - Rosenberg, M. (1965). *Society and the Adolescent Self-Image*. Princeton University Press; JSTOR. - Soto, C. J., & John, O. P. (2017). Short and extra-short forms of the Big Five Inventory–2: The BFI-2-S and BFI-2-XS. *Journal of Research in Personality*, *68*, 69–81. - Kovacs, M. (2015). Children’s Depression Inventory (CDI and CDI 2). In *The Encyclopedia of Clinical Psychology* (pp. 1–5). John Wiley & Sons, Ltd |
| Hull et al, 2021 | Description of clinical cases. | *none* |
| Ilic-Zivojinovic et al, 2023 | Self-developed questionnaire; the Internet Addiction Test; the CES-DC. | - Young, K. S., & de Abreu, C. N. (2011). *Internet addiction: A handbook and guide to evaluation and treatment* (pp. xvii, 289). John Wiley & Sons, Inc. - Weissman, M. M., Orvaschel, H., & Padian, N. (1980). Children’s symptom and social functioning self-report scales. Comparison of mothers’ and children’s reports. *The Journal of Nervous and Mental Disease*, *168*(12), 736–740. |
| López-Gil et al, 2023 | Self-developed questionnaire; the Social Network Addiction Scale (SNAddS-6S); the Sick, Control, One, Fat, Food (SCOFF) questionnaire | - Cuadrado, E., Rojas, R., & Tabernero, C. (2020). Development and Validation of the Social Network Addiction Scale (SNAddS-6S). *European Journal of Investigation in Health, Psychology and Education*, *10*(3), 763–778. - Garcia-Campayo, J., Sanz-Carrillo, C., Ibañez, J. A., Lou, S., Solano, V., & Alda, M. (2005). Validation of the Spanish version of the SCOFF questionnaire for the screening of eating disorders in primary care. *Journal of Psychosomatic Research*, *59*(2), 51–55. |
| Maes et al, 2022 | Self-developed questionnaire; the Sociocultural Attitudes Towards Appearance Questionnaire-4 (STATAQ-4); the 9-figure contour scale. | - Schaefer, L. M., Burke, N. L., Thompson, J. K., Dedrick, R. F., Heinberg, L. J., Calogero, R. M., Bardone-Cone, A. M., Higgins, M. K., Frederick, D. A., Kelly, M., Anderson, D. A., Schaumberg, K., Nerini, A., Stefanile, C., Dittmar, H., Clark, E., Adams, Z., Macwana, S., Klump, K. L., … Swami, V. (2015). Development and validation of the Sociocultural Attitudes Towards Appearance Questionnaire-4 (SATAQ-4). *Psychological Assessment*, *27*(1), 54–67. - *Stunkard, A. J., Sorensen, T., & Schulsinger, F. (1983). Use of the Danish Adoption Register for the Study of Obesity and Thinness. Research Publications—Association for Research in Nervous & Mental Disease, 60, 115-120. - References—Scientific Research Publishing*. (s.d.). Recuperato 20 aprile 2024, |
| Marengo et al, 2022 | Self-developed questionnaire; the Italian Bergen Social Media Addiction Scale developed (BSMAS). | - Monacis, L., De Palo, V., Griffiths, M. D., & Sinatra, M. (2017). Social networking addiction, attachment style, and validation of the Italian version of the Bergen Social Media Addiction Scale. *Journal of Behavioral Addictions*, *6*(2), 178–186 |
| Muñoz- Rodríguez et al, 2023 | Self-developed questionnaire; the CHAID algorithm decision tree. | *none* |
| Nagy et al, 2022 | Description of clinical cases. | *none* |
| Pruccoli et al, 2022 | Self-developed questionnaire. | *none* |
| Qin et al, 2023 | Self-developed questionnaire. | - Cao, X., Gong, M., Yu, L., & Dai, B. (2020). Exploring the mechanism of social media addiction: An empirical study from WeChat users. *Internet Research*, *30*(4), 1305–1328. - Chen, C., Zhang, K. Z. K., Gong, X., Zhao, S. J., Lee, M. K. O., & Liang, L. (2017). Understanding compulsive smartphone use: An empirical test of a flow-based model. *International Journal of Information Management*, *37*(5), 438–454. - Kim, D., & Ko, Y. J. (2019). The impact of virtual reality (VR) technology on sport spectators’ flow experience and satisfaction. *Computers in Human Behavior*, *93*, 346–356. - Nikken, P., & Jansz, J. (2006). Parental mediation of children’s videogame playing: A comparison of the reports by parents and children. *Learning, Media and Technology*, *31*(2), 181–202. - Fan, F. (2005a). A Study on the Internet Dependence of College Students: The Revising and Applying of a Measurement. *Psychological development and education*. |
| Qin et al, 2022 | Self-developed questionnaire. | - Zhang, K., Min, Q., Liu, Z., & Liu, Z. (2016). Understanding microblog continuance usage intention: An integrated model. *Aslib Journal of Information Management*, *68*(6), 772–792. - Lee, S., & Kim, B. G. (2017). The impact of qualities of social network service on the continuance usage intention. *Management Decision*, *55*(4), 701–729 - Nelson, R. R., Todd, P. A., & Wixom, B. H. (2005a). Antecedents of Information and System Quality: An Empirical Examination Within the Context of Data Warehousing. *Journal of Management Information Systems*, *21*(4), 199–235 - Cao, X., Gong, M., Yu, L., & Dai, B. (2020). Exploring the mechanism of social media addiction: An empirical study from WeChat users. *Internet Research*, *30*(4), 1305–1328. - Chen, C., Zhang, K. Z. K., Gong, X., Zhao, S. J., Lee, M. K. O., & Liang, L. (2017). Understanding compulsive smartphone use: An empirical test of a flow-based model. *International Journal of Information Management*, *37*(5), 438–454. - Novak, T., Hoffman, D., & Yung, Y.-F. (2000). Measuring the Customer Experience in Online Environments: A Structural Modeling Approach. *Marketing Science*, *19*, 22–42. - Fan, F. (2005a). A Study on the Internet Dependence of College Students: The Revising and Applying of a Measurement. *Psychological development and education*. |
| Sagrera et al, 2022 | Self-developed interview. | *none* |
| Sarman et al, 2023 | Self-developed questionnaire; the UCLA Loneliness Scale; the Adolescent Anger Rating Scale. | - Russell, D., Peplau, L. A., & Cutrona, C. E. (1980). The revised UCLA Loneliness Scale: Concurrent and discriminant validity evidence. *Journal of Personality and Social Psychology*, *39*(3), 472–480. - McKinnie Burney, D., & Kromrey, J. (2001). Initial development and score validation of the Adolescent Anger Rating Scale. *Educational and Psychological Measurement*, *61*(3), 446–460. |
| Sha et al, 2021 | Smartphone Addiction Scale short version adapted for TikTok; Depression Anxiety Stress Scales 21; forward and backward digit spans. | - Kwon, M., Kim, D.-J., Cho, H., & Yang, S. (2013). The Smartphone Addiction Scale: Development and Validation of a Short Version for Adolescents. *PLOS ONE*, *8*(12), e83558. - Ng, F., Trauer, T., Dodd, S., Callaly, T., Campbell, S., & Berk, M. (2007). The validity of the 21-item version of the Depression Anxiety Stress Scales as a routine clinical outcome measure. *Acta Neuropsychiatrica*, *19*(5), 304–310. |
| Soriano-Ayala et al, 2022 | Study 1: adapted and abbreviated version of the scientific scale of Graff et al (2013). | - Graff, K. A., Murnen, S. K., & Krause, A. K. (2013). Low-cut shirts and high-heeled shoes: Increased sexualization across time in magazine depictions of girls. *Sex Roles: A Journal of Research*, *69*(11–12), 571–582. - Braun, V., & Clarke, V. (2006). Using thematic analysis in psychology. *Qualitative Research in Psychology*, *3*(2), 77–101. |
|  | Study 2: self-developed semi-structured interview; thematic analysis of contents. |  |
| Wu et al, 2021 | Self-developed questionnaire; the Satisfaction with Life scale; the Positive Affect and Negative Affect Scale for Children. | - Diener, E., Emmons, R. A., Larsen, R. J., & Griffin, S. (1985). The Satisfaction With Life Scale. *Journal of Personality Assessment*, *49*(1), 71–75. - Laurent, J., Catanzaro, S. J., Joiner Jr., T. E., Rudolph, K. D., Potter, K. I., Lambert, S., Osborne, L., & Gathright, T. (1999). A measure of positive and negative affect for children: Scale development and preliminary validation. *Psychological Assessment*, *11*(3), 326–338. |

**JOURNAL:** [**European Child & Adolescent Psychiatry**](https://link.springer.com/journal/787)

**ARTICLE TITLE: Scrolling through Adolescence: a systematic review of the Impact of TikTok on adolescent mental health**

Giulia Conte^1a^, Giorgia Di Iorio^1a^, Dario Esposito^1^, Sara Romano^1^, Fabiola Panvino^1^, Susanna Maggi^1^, Benedetta Altomonte^1^, Maria Pia Casini ^1^, Mauro Ferrara^1,^, Arianna Terrinoni^1*^

^a^ these authors contributed equally to this work

^1^ *Department of Human Neuroscience, Unit of Child and Adolescent Neuropsychiatry, Sapienza University of Rome, Via dei Sabelli 108, 00185 Rome, Italy*

**Corresponding author***:*

Arianna Terrinoni, MD

E-mail: a.terrinoni@policlinicoumberto1.it
